# Deformities, Including Diseases of the Bones and Joints

**Published:** 1912-09

**Authors:** 


**Deformities, including Diseases of the Bones and Joints.** By A. H. Tubby, M.S., F.R.C.S. Second Edition. Two volumes. Pp. xxxi, 883; xxiv, 867. London: Macmillan and Co. Limited. 1912.

We most heartily welcome a new edition of Mr. Tubby's very valuable book on deformities, but very greatly regret that the author has adopted the plan of American writers on orthopædic surgery, and included in the new edition diseases of the bones and joints. The book is now published in two thick volumes, and the greater part of one of them is devoted to diseases of the bones and joints. We already have a most valuable book on diseases of the joints by Professor Howard Marsh, and an equally valuable one on tuberculous diseases of bones and joints by Sir Watson Cheyne, and there is therefore no need for inclusion of these subjects in Mr. Tubby's book. We do not think the portion of the book which deals with diseases of bones, other than tuberculous diseases, is at all adequate to the purpose, and had better have been omitted altogether; but the portion of the book dealing with tuberculous diseases of bones and joints, particularly the chapter on tuberculous disease of the spine, seems very complete. We notice reference to Mr. Harold Stiles's method of resecting a portion of the diaphysis for tuberculous disease, and his illustrations are reproduced from Burghard's *System of Operative Surgery*. But all the chapters on diseases of bones and joints might well have been omitted. As well might aneurysm or varicose veins or any other disease of the limbs be included. If by orthopædic surgery we understand the surgery of deformities, that is quite a reasonable sub-division of surgery. It has been, we know, suggested that all diseases of the organs of locomotion should be included. If that is so, why has not Mr. Tubby a chapter on diseases of tendons and their sheaths, and of the peripheral nerves which innervate the muscles, and why does he include such a deformity as cervical rib? The only satisfactory sub-division of surgery as orthopædic surgery is to regard it as a sub-division dealing with deformities.

One great value of the new edition of Mr. Tubby's work is its inclusion of the very valuable information contained in the book by the author and Mr. Robert Jones on the surgery of paralysis, and this information is, of course, brought well up to date. The illustrations and reproductions of skiagrams throughout the work are excellent.

In a new edition of a work on deformities we are interested to note the author's recent views on the treatment of congenital dislocation of the hip and coxa vara. Mr. Tubby quotes the published results of various surgeons on the treatment of congenital dislocation of the hip by Lorenz's manipulative method, and refers to the rapid rise in the rate of successes to failures due to improvements in technique. He is still in favour of operation in cases in which manipulation fails, but not of the Hoffa-Lorenz operation of deepening the acetabulum, only in the open reposition of the head in what there is of an acetabulum. We know from experience that this may not be possible, even when the joint is freely opened.

In the treatment of coxa vara the author advises removal of weight-bearing through the limb, by the use of a Thomas caliper splint, and "if pain or spasm" are present he then recommends weight extension. If operation has to be undertaken he is in favour of Whitman's method of removal of a wedge from just below the great trochanter.

We are glad to see that the author, in describing the pathology of genu valgum, does admit that the tibia may be markedly deformed, but he does not seem to have yet examined a sufficient number of skiagrams to be convinced how often this is so, and in how many cases the curve in the tibia is the essential cause of the genu valgum. Hence we fail to find reference to the most reasonable method of its correction—osteotomy through the deformed bone. We do not agree with the author that adolescent genu valgum is generally due to a yielding of the internal lateral ligament, with secondary change in the lower end of the femur, if bony change is present at all. We have seen many cases of adolescent genu valgum, with quite marked curve in the shaft of the tibia, and in some, but to a less degree, deformity in the lower end of the femur also, but have not come across cases due to stretched internal lateral ligament.

We turned with much interest to see what operative treatment the author recommended for congenital talipes equino-varus, as there is so much difference of opinion on the subject. We find he is not an advocate of Phelp's operation, but describes at considerable length the manipulation method of Lorenz and Wolff, which is really only manual wrenching. It seems as marvellous to us as it apparently did to Volkmann that severe cases can be corrected in this way, and we share the fear of those surgeons who think severe damage may be done to the foot if correction of the deformity is carried out at one operation.

This new edition of the book is, we think, the best work of any British surgeon on the subject, and must have involved much labour in its production. Indeed, Mr. Tubby tells us in his Preface that if he had fully appreciated the magnitude and difficulties of his task it is probable that his courage would have failed. It is fortunate for surgeons that he did not appreciate it, or we should have been without a very valuable and much enlarged edition of his book, which should be consulted by all who wish for full and recent information on the subject of deformities.

